# Jiedu Xiaozhen Granules for Epidermal Growth Factor Receptor Tyrosine Kinase Inhibitor–Mediated Skin Toxicity: Protocol for a Randomized Controlled Trial

**DOI:** 10.2196/79579

**Published:** 2026-01-22

**Authors:** Shoujiang Hao, Xiaoli Li, Shulan Hao, Xiaoying Zhang, Xiaojun Qi, Gang Jin, Fangfang Shen, Likun Liu

**Affiliations:** 1 Shanxi University of Traditional Chinese Medicine, 89 Section 1 Jinci Road, Taiyuan, China, 86 17631765869; 2 Shanxi Province Hospital of Traditional Chinese Medicine, 46 Bingzhou West Street, Yingze District, Taiyuan, Shanxi, 030012, China, 86 17631765869; 3 The Second Affiliated Hospital of Shanxi Medical University, Taiyuan, China; 4 Shanxi Provincial Cancer Hospital, Taiyuan, China

**Keywords:** traditional Chinese medicine, non–small cell lung cancer, targeted drugs, jiedu xiaozhen granules, skin toxicity

## Abstract

**Background:**

Epidermal growth factor receptor tyrosine kinase inhibitors (EGFR-TKIs) are widely used in the treatment of non–small cell lung cancer due to their precision, efficiency, and ease of use. However, skin rashes induced by EGFR-TKIs are the most common and earliest form of skin toxicity, often affecting the quality of life and treatment compliance of patients and leading to early discontinuation of therapy. These skin reactions may even impact cancer outcomes. In clinical practice, traditional Chinese medicine detoxification granules have shown effectiveness in relieving skin discomforts such as itching, pain, and burning caused by EGFR-TKI therapy. A prior single-arm trial investigating the treatment of targeted drug–induced rashes demonstrated a sustained improvement in rash symptoms with an effectiveness rate of 80.77% and was well tolerated by patients.

**Objective:**

As an exploratory clinical study, this randomized controlled trial will preliminarily evaluate the potential efficacy and safety of jiedu xiaozhen (JDXZ) granules in managing EGFR-TKI–related skin toxicities.

**Methods:**

This randomized controlled trial will be conducted at Shanxi Provincial Hospital of Traditional Chinese Medicine. A total of 94 patients with confirmed epidermal growth factor receptor gene–mutated non–small cell lung cancer who developed rashes after EGFR-TKI treatment will be enrolled. Patients will be randomly assigned to either a JDXZ traditional Chinese medicine group (group J) or a urea ointment group (group U). The primary outcome will be the severity of the rash as assessed using the National Cancer Institute Common Terminology Criteria for Adverse Events grading. Secondary outcomes will include the WoMo (Wollenberg and Moosmann) score, numerical rating scale, Dermatology Life Quality Index scale, European Organisation for Research and Treatment of Cancer Quality of Life Questionnaire Core 30 score, median progression-free survival, and changes in the levels of fibroblast growth factor 7 and hepatocyte growth factor in the blood. Adverse reactions will be recorded throughout the study. Data will be analyzed using SPSS.

**Results:**

The clinical trial registration was completed in October 2024. This study is currently underway. As of December 1, 2025, a total of 81 eligible participants had been enrolled, all of whom were assigned to groups following the randomization principle. Among them, 42 participants were allocated to the JDXZ group (with an additional 2 participants pending enrollment), and 39 to the control group. Based on the current progress, the estimated trial completion date has been extended to January 31, 2026.

**Conclusions:**

The results of this study may help develop an effective treatment for EGFR-TKI–mediated rashes. The findings will be published in academic journals upon the completion of the trial.

## Introduction

Patients with non–small cell lung cancer (NSCLC) who harbor epidermal growth factor receptor (*EGFR*) mutations represent a unique subgroup [1]. Women, nonsmokers, and patients with adenocarcinoma are more likely to carry *EGFR* mutations, with an incidence of 30% to 35% in East Asian populations [2]. In the Chinese NSCLC population, the prevalence of *EGFR* mutations is 28.2%, rising to 50.2% in those with lung adenocarcinoma [3]. *EGFR* tyrosine kinase inhibitors (EGFR-TKIs) selectively block the *EGFR* signaling pathway, thereby improving survival in patients with NSCLC carrying *EGFR* mutations [4]. It is categorically endorsed as a grade IA recommendation for the postoperative, locally advanced, and advanced first-line treatment of EGFR-positive NSCLC [5]. However, as *EGFR* is also expressed in skin keratinocytes, sebaceous glands, hair follicles, and perinuclear tissues [6], inhibiting *EGFR* activity can trigger a cascade of intracellular signaling pathways. The integrity of the human epidermis is compromised, the proliferation of epidermal cells is perturbed, the sebaceous gland lipid secretion is disrupted, and the skin’s hydration retention function is impaired, culminating in the manifestation of adverse cutaneous reactions [7].

According to phase 3 clinical trials approved by the US Food and Drug Administration, the incidence of skin rash associated with gefitinib use was 66% in patients from East Asia, whereas 3.1% experienced grade 3 rashes. Erlotinib use resulted in a 73% rash incidence, with 32% being grade 3; afatinib use led to rashes in 80.8% of patients, with 14.6% experiencing grade 3 rashes. In China, icotinib use caused rashes in 15.5% of patients, with 14.9% experiencing grade 3 rashes [8]. Results from the FLAURA2 trial found that the incidence of grade 3 adverse effects was higher in the combination therapy group than in the monotherapy group (ranging from 11% to 54%), with serious adverse effects increasing from 5% to 19%. Osimertinib, another EGFR-TKI, was associated with skin toxicity in approximately 24% of patients [9].

The most common and earliest cutaneous side effects of anti-*EGFR* therapies are papules and pustules distributed in seborrheic areas such as the face, scalp, and upper trunk, sometimes extending to the limbs, palms, and soles. These reactions typically occur within 1 to 2 weeks of starting targeted therapy and are dose dependent. It is noteworthy that this issue is encountered in a minimum of 75% of the patient population [10]. The severity of the rash is often positively correlated with the response to treatment of the patients [11]. Approximately 35% of patients initially manifest dry, pruritic dermatitis affecting the extremities [12,13]. The onset of sebum deficiency in eczema is typically concurrent with the exacerbation of the condition, culminating in a pustular rash secondary to either *Staphylococcus aureus* or herpes simplex infection [14], which may or may not be accompanied by hyperpigmentation and telangiectasia [15]. Unlike acne vulgaris [16], these skin reactions are characterized by erythematous papules and pustules without acne or cysts. Other associated symptoms include dry skin, burning sensations, itching, pain, and changes to the hair and nails (eg, paronychia) [17]. To manage these side effects, patients should avoid contact with irritants, particularly in areas prone to rashes. Daily skin care should include the use of moisturizers and sun protection and the avoidance of extreme temperatures. Wearing comfortable clothing and shoes and using nonirritating bath products is also recommended [18]. It is imperative to mitigate the adverse cutaneous reactions that are elicited.

At present, the assessment of targeted drug–induced cutaneous toxicities is conducted in accordance with the 2017 National Cancer Institute Common Terminology Criteria for Adverse Events (NCI-CTCAE). Various guidelines, including those from the Multinational Association of Supportive Care in Cancer skin toxicity study group, the National Comprehensive Cancer Network, and the European Society for Medical Oncology, offer different expert opinions and consensuses on managing skin toxicity [11,19-21]. However, these guidelines lack comprehensive, evidence-based recommendations [8,10]. As patients often experience dry, itchy skin that compromises their skin barrier, most treatment strategies focus on symptom relief, and there are discrepancies in clinical practice regarding medication use [8,10].

The dermatological adverse effects associated with *EGFR* inhibitors are transient and tend to diminish with extended treatment duration. Resolution of the rash is typically complete upon cessation of therapy, and it is seldom constrained by the dosage or duration of treatment. Xerosis and pruritus are commonly attributable to a compromised skin barrier function. The predominant therapeutic modalities used for the management of grade 1 or 2 cutaneous toxicity encompass topical treatments such as 2.5% hydrocortisone cream, topical antibiotics (including erythromycin cream, clindamycin emulsion, metronidazole cream, and tacrolimus cream), and moisturizers (such as urea cream and fusidic acid cream). For more severe cases, oral medications may be considered, such as doxycycline (100 mg twice daily) or minocycline (50‐100 mg twice daily). Oral antihistamines, including levocetirizine (5 mg daily), desloratadine (5 mg daily), diphenhydramine (25‐50 mg 3 times daily), hydroxyzine (25 mg 3 times daily), or fexofenadine (60 mg 3 times daily), can help manage pruritus. For grade 3 rashes, oral tretinoic acid (0.3‐0.5 mg per kilogram) or prednisone (0.5 mg per kilogram) may be prescribed. Gamma-aminobutyric acid receptor agonists such as gabapentin (300 mg every 8 hours) or pregabalin (50‐75 mg every 8 hours) can also be considered for symptom relief. In severe cases, the dosage of targeted drugs should be reduced according to clinical guidelines, and for grade 4 rashes, oral steroid use may be escalated, and targeted therapy should be discontinued as soon as possible. Additionally, other studies have explored alternative treatments. For example, a study by Nagase et al [22] found that rapid desensitization therapy was effective in treating severe rashes caused by molecularly targeted drugs in patients with NSCLC. Furthermore, vitamin K_1_ has shown potential in preventing rashes associated with *EGFR* inhibitors, possibly reducing both the incidence and severity of acne-like rashes [18,21].

Retinoic acid drugs are commonly used in clinical practice, but higher doses can exacerbate skin and mucosal dryness. Additionally, due to their photosensitivity, patients need to avoid radiation exposure as these factors can worsen rashes [23]. Doxycycline has been found to reduce the severity of rashes induced by EGFR-TKIs [18]. However, in clinical practice, doxycycline and minocycline are not frequently used for treatment; instead, they are primarily used in preventive studies [18,24-26]. The findings of Deplanque et al [25] also confirmed that, while doxycycline may not lower the incidence of erlotinib-induced folliculitis, it does reduce the severity of the rash. It is important to note the potential side effects of tetracycline antibiotics, which include anorexia, nausea, vomiting, diarrhea, esophagitis, and esophageal ulcers [27]. Systemic steroid hormones are usually not the first line of treatment for *EGFR* inhibitor–associated rashes due to their own side effects [19], such as endocrine disorders, osteoporosis, and weakened immunity, which can have serious consequences [28].

Due to the complex nature of infections and the standardized use of antibiotics, it is important to remain vigilant about antibiotic resistance. Glucocorticoids, while potent in treating skin toxicity, should only be considered when other treatment options are ineffective, making both antibiotic therapy and glucocorticoid therapy less ideal for routine clinical use. Patients receiving molecular-targeted therapies are often highly sensitive to irritants and allergens, complicating clinical treatment decisions. Most of the patients with lung cancer and *EGFR* mutations who experience cutaneous adverse reactions to EGFR-TKIs do not seek treatment due to a lack of awareness of or confidence in existing treatment options. Research reveals that EGFR-TKI skin toxicity stems from a compromised skin barrier linked to abnormal keratinocyte differentiation, apoptosis, and inflammatory infiltration [29]. To guarantee the rational deployment and therapeutic efficacy of clinical pharmacotherapy, it is advisable to use mild emollients, which incorporate active constituents such as urea, ceramide, plant sterols, and linolenic acid, among others, for the purpose of local hydration; moisturization; anti-inflammatory action; antipruritic effects; and amelioration of cutaneous symptoms such as dryness, itching, and pain [29]. Consequently, the identification of novel and well-tolerated therapeutic modalities to address drug-induced dermatotoxicity continues to pose a significant challenge for the oncological community.

Traditional Chinese medicine (TCM) active ingredients and preparations are increasingly used in skin disease treatment as modern pharmacology and clinical trials advance [30]. Jiedu xiaozhen (JDXZ) granules, a blend of *Astragalus membranaceus*, *Isatis indigotica Fort*, and *Concha margaritifera*, show promise in managing EGFR-TKI–induced skin toxicity. Author LL, with 40 years’ experience in TCM, has shown JDXZ granules’ efficacy and safety for treating EGFR-TKI–related skin toxicity. The oral JDXZ granules in their single-arm trial [31] effectively alleviated systemic symptoms, sustaining rash improvement and reducing posttargeting recurrence with minimal side effects. In clinical observation, targeted drug–related skin toxicity showed sustained improvement with an effectiveness rate of 80.77% and was well tolerated by patients. This exploratory randomized controlled trial (RCT) will preliminarily evaluate the efficacy and safety of JDXZ granules to explore a potential management strategy for skin toxicity induced by EGFR-TKIs.

## Methods

### Study Design

**Figure 1.**
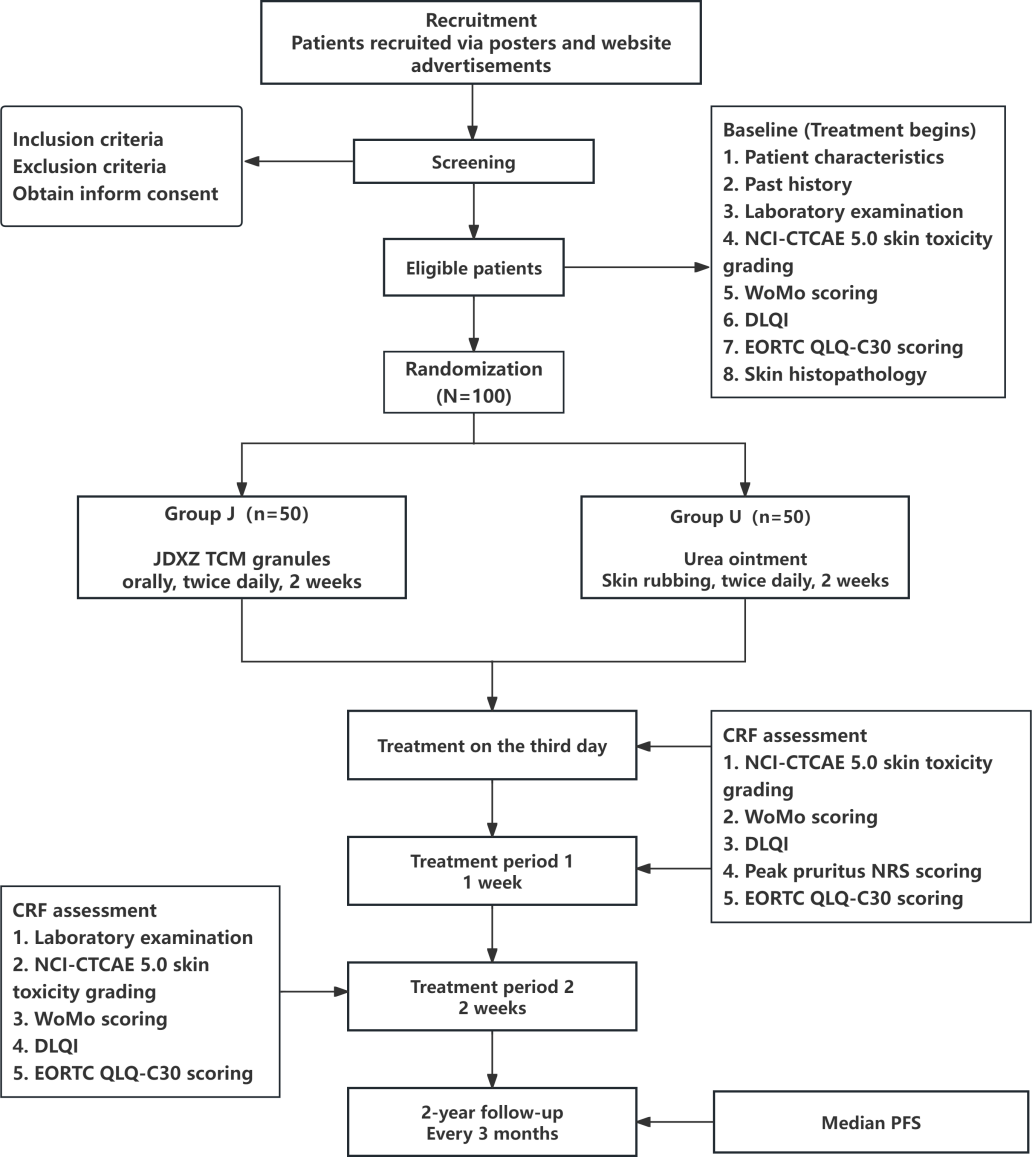
Clinical trial flowchart. CRF: case report form; DLQI: Dermatology Life Quality Index; EORTC QLQ-C30: European Organisation for Research and Treatment of Cancer Quality of Life Questionnaire Core 30; JDXZ: jiedu xiaozhen; laboratory examination: changes in blood levels of factors such as fibroblast growth factor 7 and hepatocyte growth factor; NCI-CTCAE: National Cancer Institute Common Terminology Criteria for Adverse Events; NRS: numerical rating scale; PFS: progression-free survival (time during which patients remain free from disease progression; follow-up every 3 months); TCM: traditional Chinese medicine; WoMo (Wollenberg and Moosmann) score: ranges from 0 to 100 and is calculated using the formula final score=1/4A + 1/4B + 10/3C).

This study is a multicenter, prospective, randomized controlled clinical trial in which participants will receive either enhanced TCM or positive control drugs. The trial will involve 94 patients with a confirmed diagnosis of *EGFR* gene mutation–positive NSCLC who developed skin rashes after using EGFR-TKIs. The participants will be divided into 2 groups: the treatment group (receiving oral JDXZ granules) and the control group (receiving topical urea ointment). Effectiveness will be assessed by comparing baseline conditions with the results at 2 weeks after treatment. Each patient will undergo 4 scheduled visits: at baseline and on days 3, 7, and 14 (Figure 1).

### Study Setting

Participants will be recruited from 3 centers: Shanxi Provincial Hospital of Traditional Chinese Medicine, Shanxi Cancer Hospital, and the Second Affiliated Hospital of Shanxi Medical University. Adequate sample sizes will be obtained through various hospital departments and online platforms. Inclusion criteria will be verified, ensuring that participants have relatively complete medical records and have signed the informed consent form.

### Participants

#### Recruitment Strategy

Patients will be enrolled from 3 clinical centers, most of which have been designated as affiliated hospitals of the Lung Cancer Mutation Consortium. Recruitment advertisements will be posted in hospital bulletins, social media, and medical forums. The enrollment procedure will be streamlined to facilitate expeditious and hassle-free completion by the patients, thereby mitigating potential obstacles to their participation.

#### Eligibility Criteria

##### Inclusion Criteria

Inclusion criteria are as follows: (1) patients diagnosed with NSCLC carrying *EGFR* gene mutations who have developed skin rashes following EGFR-TKI treatment, with rash severity graded as 1 to 3 according to the NCI-CTCAE 5.0 standards; (2) patients aged ≥18 years, with no gender restrictions, and who have a Karnofsky Performance Status score of at least 60; (3) patients with adequate hematological, hepatic, renal, and metabolic function as indicated by specific blood count and organ function parameters (white blood cells of >3 × 10^9^ per liter, absolute neutrophil count of ≥1.5 × 10^9^ per liter, platelet count of ≥100 × 10^9^ per liter, hemoglobin of >9 g/dL, serum creatinine of ≤1.5 times the upper limit of normal [ULN], bilirubin of ≤1.5 times the ULN, aspartate aminotransferase or alanine transaminase of ≤2.5 times the ULN, if liver metastases are present, AST/ALT ≤ 5 × ULN, and serum electrolyte within normal range); and (4) patients who are willing to receive the study treatment and sign the informed consent form with good compliance.

##### Exclusion Criteria

Exclusion criteria are as follows: (1) impaired visceral function; (2) a history of significant cardiovascular diseases (including myocardial infarction, unstable angina pectoris, symptomatic congestive heart failure, and severe uncontrolled arrhythmia) within the previous year, interstitial lung disease (such as pneumonia or pulmonary fibrosis), or evidence of interstitial lung disease on a baseline chest computerized tomography scan; (3) concurrent antitumor treatments; (4) rashes unrelated to EGFR-TKI drugs or recent rash-related treatments; (5) participation in other clinical trials within 4 weeks prior; (6) diagnosis of dialectical spleen or stomach deficiencies or cold syndromes as per TCM; (7) known allergies to trial medications; and (8) pregnant or lactating women and those planning to become pregnant within 6 months after the end of treatment.

##### Withdrawal Criteria

Patients will be withdrawn from the study if they experience serious adverse events, such as nausea, vomiting from oral TCM, or liver and kidney insufficiency, making further observation unsuitable. Additionally, patients who fail to complete the treatment and observation period outlined in the protocol for any reason will be considered for withdrawal. Patients unwilling to continue treatment and requesting to exit the trial will also be withdrawn. Finally, any disease progression that affects or interferes with the study outcomes will result in the withdrawal of the patient from the trial.

### Randomization and Blinding

The randomization sequence will be generated by the data manager. The patients will be randomly assigned to either the treatment group or the control group in a 1:1 ratio. Blinding methods will not be used in this clinical trial. Although blinding of participants is not feasible due to the nature of the intervention, the following blinding strategies will be implemented in the study design to minimize potential bias: health care providers administering the treatment, outcome assessors, and data analysts will all be kept blinded to group allocation. Specifically, the treatment providers responsible for assigning the interventions will not participate in subsequent efficacy or safety evaluations. During follow-up visits, clinical assessors will perform dermatological lesion observation and grading without knowledge of the participants’ group assignments. For the final statistical analysis, analysts will process data using anonymized codes, and blinding will only be lifted after the statistical analysis is completed. This approach was designed to ensure objectivity and accuracy in outcome evaluation and data interpretation.

### Interventions

In the treatment group, after EGFR-TKI treatment, patients will receive daily oral TCM-based granule treatment. The prescription consists of *A membranaceus* (30 g), *I indigotica Fort* (15 g), and *C margaritifera* (15 g). The granules will be taken twice daily, in the morning and evening, following meals for 7 days per treatment course, with 2 consecutive courses administered. In the control group, patients will apply the urea ointment to the affected areas daily, 1 to 3 times per day, with each course lasting 7 days, also for 2 consecutive courses. All medications were supplied by Shanxi Provincial Hospital of Traditional Chinese Medicine. Investigators will assess patients on days 0, 3, 7, and 14 (Table 1). During the observation period, all patients will avoid contact with irritants, particularly in areas prone to rash, and will use sunscreen daily to reduce the incidence of skin rashes.

**Table 1.** Schedule of the jiedu xiaozhen granule clinical trial.

	Visit 0 (day 0; ±2 days)	Visit 1 (day 3; ±2 days)	Visit 2 (day 7; ±2 days)	Visit 3 (day 14; ±2 days)	Visit 4 (3, 6, 9, 12, 15, 18, 21, and 24 months)
Signature of the informed consent form	✓				
Inclusion and exclusion criteria for verification	✓				
Randomization grouping	✓				
Release of experimental drugs	✓				
Recovery of the experimental drugs				✓	
Basic data collection					
Demographic data	✓				
Personal history and allergy history	✓				
Study disease–related history	✓				
Evaluation of the condition and its effectiveness^a^	✓	✓	✓	✓	
History of other diseases	✓				
Concomitant medications or treatment	✓	✓	✓	✓	
Security observations					
Liver and kidney function examination	✓			✓	
Untoward effect		✓	✓	✓	
Efficacy observation					
Clinical efficacy rate (NCI-CTCAE^b^ version 5.0)	✓	✓	✓	✓	
WoMo score	✓	✓	✓	✓	
Peak pruritus NRS^c^	✓	✓	✓	✓	
DLQI^d^	✓	✓	✓	✓	
EORTC QLQ-C30^e^ scale (quality of life measurement)	✓	✓	✓	✓	
Median PFS^f^					✓
Laboratory examination (FGF7^g^ and HGF^h^ in human blood)	✓	✓		✓	
Skin histopathological examination	✓				
Evaluation of medication compliance		✓	✓	✓	
Adverse events and complications		✓	✓	✓	
Serious adverse events		✓	✓	✓	

a A systematic and comprehensive evaluation of patients with non–small cell lung cancer was conducted in accordance with the Response Evaluation Criteria in Solid Tumors (RECIST 1.1), encompassing changes in pulmonary lesions and metastatic foci, as well as indicators such as tumor marker.

b NCI-CTCAE: National Cancer Institute Common Terminology Criteria for Adverse Events.

c NRS: numerical rating scale.

d DLQI: Dermatology Life Quality Index.

e EORTC QLQ-C30: European Organisation for Research and Treatment of Cancer Quality of Life Questionnaire Core 30.

f PFS: progression-free survival.

g FGF7: fibroblast growth factor 7.

h HGF: hepatocyte growth factor.

To uphold the integrity of the study, participants are permitted to engage in fundamental skin care regimens throughout the trial and will maintain diligent sun protection measures and refrain from indulging in excessive cleansing or the application of abrasive cosmetics on a daily basis. Nevertheless, it is imperative to abstain from the ingestion of any other pharmacological agents or the implementation of interventions that could potentially disrupt the therapeutic process for the rash, encompassing, though not restricted to, hormonal medications and immunosuppressants. Should any significant cutaneous discomfort or additional complications arise, it is essential to promptly notify the attending physician for a timely evaluation and modification of the treatment plan if necessary.

### Assessments

#### Primary Outcomes

In conformity with the NCI-CTCAE version 5.0 and the efficacy assessment criteria in alignment with the *Guiding Principles for Clinical Research on New Chinese Medicine Drugs* [32], the criterion for improvement was defined as a posttreatment rash grade of 0, denoting complete recovery. A ≥2-grade improvement in the rash classification was defined as a marked effect. A single-grade improvement in the rash classification was deemed effective. Should these criteria not be satisfied and the rash either remain unaltered in severity or worsen by more than 1 grade, the treatment will be regarded as ineffective. The following formula will be used: clinical efficacy rate=(cured + significantly improved + improved cases)/total cases × 100% .

#### Secondary Outcomes

The secondary outcomes include the WoMo (Wollenberg and Moosmann) score, which ranges from 0 to 100 and is calculated using the formula final score= 1/4A + 1/4B + 10/3C. Acnelike rash scores of 0 to 20 are considered mild, scores of 21 to 40 are considered moderate, and scores of 41 to 100 are considered severe (Multimedia Appendix 1). Other outcomes include the peak pruritus numerical rating scale, which measures the intensity of pruritus; the dermatology Life Quality Index, which assesses the impact of skin disease on quality of life; the European Organisation for Research and Treatment of Cancer Quality of Life Questionnaire Core 30 scale (quality of life measurement), which evaluates overall quality of life in patients with cancer; median progression-free survival, which represents the time during which patients remain free from disease progression (follow-up every 3 months); a laboratory examination to measure changes in blood levels of factors such as fibroblast growth factor 7 and hepatocyte growth factor, and a skin histopathological examination to evaluate skin tissue changes after the treatment.

#### Adverse Events

In conformity with the criteria delineated by the Ministry of Health of China’s Adverse Drug Reaction Monitoring Center, as documented in the *Guiding Principles for Clinical Research of New Chinese Medicine Drugs*, the potential relationship between adverse events and the use of the investigational drug will be appraised using a 5-tiered categorization scheme encompassing “certain,” “probable,” “possible,” “remote,” and “unrelated.” The specific evaluation metrics are meticulously outlined in the adverse event table, which is an integral component of the study’s records (Multimedia Appendix 2).

Researchers must prompt patients to honestly report medication effects, steer clear of suggestive questions, and document any adverse reactions on the adverse event form. Follow-up, including detailed records and laboratory test normalization, should continue until symptoms resolve via hospital visits, outpatient checks, or other methods based on reaction severity (Multimedia Appendix 2).

Upon detecting adverse reactions, the researchers will diagnose, treat, and decide whether to halt observation based on the condition. For serious events, the study unit must act promptly to ensure participant safety. Researchers will submit the serious adverse event report form to the oncology department, the ethics committee, and all participating units of the hospital within 24 hours, signing and dating it.

### Data Collection and Management

Informed consent will be enhanced by detailing the study’s purpose, procedure, potential discomfort, and risks, ensuring voluntary participation. Effective physician-patient communication will be fostered, promptly addressing patient needs and resolving treatment issues to boost compliance. A follow-up plan will be developed to minimize participant burden, and phones and SMS text messages, among other tools, will be used to increase follow-up rates.

Data will be collected and recorded in a case report form by trained researchers. Baseline assessment data will be collected on the sociodemographics of the patients (including gender, age, educational level, religion, marital status, occupation, income, economic burden of illness, caregiver during illness, and smoking and drinking history), as well as disease duration and any previous treatment with TCM. Medical records of clinical trial participants, both inpatient and outpatient, should be stored at the hospital to create an electronic case database.

Researchers must document medical records concurrently with treatment to ensure timeliness, completeness, and accuracy. Any corrections should be underlined and dated by the researchers without altering the original entry. The CRF Questionnaire and Adverse Event Recording Survey, completed by both the patient and physician, adheres to objectivity and sincerity. Objectivity pertains to neutral instrument design and data authenticity, mitigating researcher bias. Respondent sincerity reflects truthful participation, minimizing social desirability bias. Their synergy ensures data reliability and study validity. Value ranges and logic will be regularly verified. For uncertainties, the question list will be completed, and researchers will address the listed queries. Main concerns include the standardization of the components of Chinese herbal preparations and their quality; potential interference from concomitant medications; and possible subjectivity in skin toxicity assessment indicators. Upon finishing the data verification report, the database will be secured. Quality control documents will be kept, including records of data consistency checks, value range and logic verifications, blind audits, and researcher communications.

### Statistical Analysis

Data will be analyzed using SPSS Statistics for Windows (version 26.0; IBM Corp). Continuous data following a normal distribution will be expressed as means and SDs and compared within groups using a paired 2-tailed *t* test or between groups using an independent-sample 2-tailed *t* test. Data will be presented as frequencies and percentages, with chi-square tests for group comparisons and rank sum tests for ranked data. For continuous data that are not normally distributed, between-group comparisons will be performed using the Mann-Whitney *U* test, with data described as medians and IQRs. For measurements involving multiple time points, nonparametric methods such as the Friedman test will be used to ensure robust and reliable findings. A *P* value of <.05 will be considered statistically significant.

### Sample Size

On the basis of a small-sample clinical trial, the effectiveness rate of the treatment group was 80.77% [31]. Additionally, a previous randomized controlled double-blind clinical study had a response rate of 45% [33]. Using the PASS tool (version 21), with a significance level of .05 and a power of 0.9, the total sample size was calculated as 78 participants. Accounting for a 20% dropout rate, the required sample size was determined to be 94 patients, with 47 in each group.

### Ethical Considerations

This RCT has received joint ethics approval from the institutional review boards of Shanxi Provincial Hospital of Traditional Chinese Medicine, Shanxi Cancer Hospital, and the Second Affiliated Hospital of Shanxi Medical University (approval 2024 KY-07026 and 2024KYY1-07014). Participants who sign the informed consent form will be enrolled in the study, with the right to withdraw at any point during the trial. Finally, the results of this RCT will be published in a relevant academic journal on completion of the trial irrespective of whether the RCT yields negative or positive outcomes. This study has been registered with the Chinese Clinical Trial Registry (ChiCTR2400086657).

### Trial Monitoring

Paper details will be managed by a designated individual and stored securely. Access to the e–medical case system will be restricted to authorized users. Before the commencement of the project, the researchers underwent rigorous training on the experimental protocol, and the uniformity of the quantitative metrics for symptoms and signs was meticulously verified. Any modifications to this protocol are subject to the prior approval of the ethics committee at Shanxi Provincial Hospital of Traditional Chinese Medicine to ensure the trial’s probity and adherence to ethical standards.

## Results

This study is currently underway. As of December 1, 2025, a total of 81 eligible participants had been enrolled, all of whom were assigned to groups following the randomization principle. Among them, 42 participants were allocated to the JDXZ group (with an additional 2 participants pending enrollment), and 39 to the control group. Based on the current progress, the estimated trial completion date has been extended to January 31, 2026.

## Discussion

Currently, the investigation into skin-related toxicities caused by EGFR-TKIs remains in the exploratory phase, with its findings yet to be fully substantiated, thereby necessitating further comprehensive research. EGFR-TKIs have the potential to elicit the aggregation of inflammatory cells within the context of normal skin. A study by Satoh et al [34] showed EGFR-TKIs’ synergy with skin *Bacillus* acnes to boost interleukin (IL)-36γ in keratinocytes. EGFR-TKIs suppressed *EGFR* signaling and upregulated KLF4, whereas *Bacillus* acnes triggered the activation of the nuclear factor kappa-light-chain-enhancer of activated B cells (NF-κB). KLF4 and NF-κB collaborate to upregulate IL-36γ promoter activity, thereby inducing neutrophil recruitment via IL-8 and worsening skin inflammation. EGFR-TKIs not only suppress the proliferative activity of basal keratinocytes but also facilitate their transition toward terminal differentiation, ultimately leading to xerosis of the skin. Erlotinib, as demonstrated by Ikarashi et al [35], markedly lowered aquaporin-3 expression in keratinocytes via the extracellular signal–regulated kinase pathway. Aquaporin-3 is crucial for skin hydration; reduced expression may impair this function. EGFR-TKIs have the potential to disrupt the intimate associations between basal keratinocytes, consequently impairing the epidermal barrier’s functionality. Gefitinib impairs the epidermal barrier by reducing claudin-1 and claudin-4 expression and increasing claudin-2 levels, as discovered by Fang et al [36].

Astragaloside IV, the main active compound of Astragalus, can inhibit the production of pro-inflammatory cytokines and the expression of TLR4 and its downstream signaling molecules, NF-κB, iNOS (inducible nitric oxide synthase), and COX-2 (cyclooxygenase-2) proteins [30]. *Astragalus* polysacharin, a monomeric *Astragalus* extract, curbs inflammatory cytokine (tumor necrosis factor, IL-6, and monocyte chemoattractant protein 1) production in lipopolysaccharide-activated RAW264.7 macrophages through NF-κB and mitogen-activated protein kinase (extracellular signal–regulated kinase and c-Jun N-terminal kinase) pathways [37]. Compounds 4 (3H) -quinazolinone (I) and 2,4 (1H, 3H) -quinazolindione (II) from purified *I indigotica Fort* effectively eliminate *S aureus*, *Escherichia coli*, *Bacillus subtilis*, and *Salmonella* [38]. Min et al [39] found that *I indigotica Fort* extract eases atopic dermatitis–like inflammation by suppressing NF-κB pathway–induced cytokine and chemokine production. Chymotrypsin severs desmosome links in skin keratinocytes, speeding cell shedding and boosting epithelial metabolism [40]. *C margaritifera*’s bioactives boost chymotrypsin, stimulate skin cell growth, and enhance collagen synthesis for skin regeneration and elasticity [40]. Reports confirm the synergistic effects of *A membranaceus*, *I indigotica Fort*, and *C margaritifera*, deeming JDXZ granules safe and effective for inflammation, antibacterial protection, and skin protection.

In the treatment of NSCLC with EGFR-TKIs, the presence of a rash has been shown to significantly predict the effectiveness of the treatment, particularly in patients with unknown *EGFR* mutation status [17]. As a result, clinicians must closely monitor skin toxicity throughout the entire course of treatment and manage it appropriately to prevent treatment discontinuation or dose reduction of EGFR-TKIs. Building on the clinical promise of JDXZ granules for EGFR-TKI-induced skin toxicity demonstrated in this RCT, a follow-up RCT is warranted to rigorously assess the treatment’s efficacy and safety, with the goals of reducing skin toxicity and enhancing patient quality of life. In this study, the severity of the rash will be graded according to the NCI-CTCAE 5.0 criteria. Assessment tools such as the itch numerical rating scale, the Dermatology Life Quality Index, and the European Organisation for Research and Treatment of Cancer Quality of Life Questionnaire Core 30 will be used. Additionally, serum levels of fibroblast growth factor 7 [41] and hepatocyte growth factor [42] will be incorporated as biomarkers, with WoMo [43,44] scores and dermatopathological findings serving as secondary end points. This approach provides a more comprehensive and updated perspective on EGFR-TKI–associated cutaneous toxicities.

However, this nonblinded study has limitations, first, in its level of evidence, with potential issues such as unevenly designed control groups and subjective bias in patient-reported outcomes (eg, itching and pain). Furthermore, the collection of long-term medication safety data remains insufficient. Should this study show promising signs, a more rigorously designed multicenter, large-sample, randomized, double-blind, double-dummy, active- or placebo-controlled clinical trial will be necessary for further validation. Such a step is critical to elevate the findings to a higher evidence level and support their potential inclusion in clinical guidelines. Future research should also focus on identifying predominant responders to JDXZ granules based on specific TCM syndrome types or biomarkers, enabling more precise application. Additionally, exploring synergistic effects with conventional Western treatments (such as topical corticosteroids or oral antibiotics) may help reduce the dosage and duration of antibiotic or corticosteroid use, thereby minimizing potential side effects.

## Supplementary material

10.2196/79579 Multimedia Appendix 1 The WoMo (Wollenberg and Moosmann) rating scale.

10.2196/79579 Multimedia Appendix 2 Instructions for adverse events.

## Abbreviations

EGFR: epidermal growth factor receptor
EGFR-TKI: epidermal growth factor receptor tyrosine kinase inhibitor
IL: interleukin
JDXZ: jiedu xiaozhen
NCI-CTCAE: National Cancer Institute Common Terminology Criteria for Adverse Events
NF-κb: nuclear factor kappa-light-chain-enhancer of activated B cells
NSCLC: non–small cell lung cancer
RCT: randomized controlled trial
TCM: traditional Chinese medicine
ULN: upper limit of normal
WoMo: Wollenberg and Moosmann
