# Supplementary material for: Jiedu Xiaozhen Granules for Epidermal Growth Factor Receptor Tyrosine Kinase Inhibitor–Mediated Skin Toxicity: Protocol for a Randomized Controlled Trial
Source: JMIR Res Protoc. 2026 Jan 22;15:e79579. doi: 10.2196/79579 (PMC12826649; doi:10.2196/79579)
Supplement: Multimedia Appendix 1 [file resprot-v15-e79579-s001.docx]

| **Table 2. The WOMO rating scale** | | | | |
| --- | --- | --- | --- | --- |
|  | | **Area** | | **Score** |
| **WoMoA** | **Degree of systemic involvement** |  | |  |
| **Annotation:** The extent of injury was expressed as a percentage (0-100%) of total body surface area (TBSA) affected, calculated using the Rule of Nines. In this method, the TBSA is divided into sections, each representing 9% or a multiple thereof: the head and neck account for 9% (3% each for scalp, face, and neck); both upper limbs constitute 18% (7% for each upper arm, 6% for each forearm, and 5% for each hand); the trunk, including the perineum, accounts for 27% (13% for the anterior trunk, 13% for the posterior trunk, and 1% for the perineum); and both lower limbs (including buttocks) account for 46% (5% for each buttock, 21% for each thigh, 13% for each lower leg, and 7% for each foot). For females, the percentage for each buttock and each foot is adjusted to 6%. | | | | |
| **WoMoB** | **Degree of facial involvement** |  | |  |
| **Annotation:** The severity of facial lesions is quantified on a scale from 0% to 100%. To more accurately discern subtle involvement, the percentage of affected facial surface area is individually assessed and documented, theoretically yielding values from 0 to 100. | | | | |
|  | | **Score 0 - 3 points** | | **Score** |
| **WoMoC** | **The depth of the erythema color** | **None** |  |  |
|  |  | **Light red** |  |  |
|  |  | **Moderate** |  |  |
|  |  | **Deep red** |  |  |
|  | **Distribution density of erythema** | **None** |  |  |
|  |  | **Scattered** |  |  |
|  |  | **Middle** |  |  |
|  |  | **Dense** |  |  |
|  | **The number, size and discoloration of papules and pustules** |  | |  |
|  | **The hardness, size and extent of spread of scales/crusts** |  | |  |
| **Annotation:** Intermediate values such as 0.5, 1.5 and 2.5 are allowed. | | | | |
| **Total (The final score ranges from 0 to 100 and is calculated using the following formula: Final score = 1/4A + 1/ 4B + 10/3C)** | | | |  |
