# Supplementary material for: Jiedu Xiaozhen Granules for Epidermal Growth Factor Receptor Tyrosine Kinase Inhibitor–Mediated Skin Toxicity: Protocol for a Randomized Controlled Trial
Source: JMIR Res Protoc. 2026 Jan 22;15:e79579. doi: 10.2196/79579 (PMC12826649; doi:10.2196/79579)
Supplement: Multimedia Appendix 2 [file resprot-v15-e79579-s002.docx]

**Instructions for adverse events**

AE name: Please refer to the specifications in the description. In addition, do not report an adverse event outcome as an adverse event. For example, "death" is the outcome of an adverse event, and the cause of death is the name of the adverse event. However, death can be reported directly when the cause of death is not known.

Medical term records in adverse events may be recorded according to the following provisions:

1. The use of a single symptom or sign name, e. g. (1) "nausea and vomiting" requires the separate recording of two adverse events, namely "nausea" and "vomiting".(2) When the investigator can make a clear diagnosis according to the relevant symptoms or signs (note the non-prediagnosis, such as urinary tract infection), report adverse events in the diagnostic name, and never report adverse events as a single symptom or sign.(3) Abnormal indicators of laboratory examination. When a clear diagnosis cannot be made when the adverse events are reported, abnormal single laboratory examination indicators (such as urinary occult blood and urinary protein Positive, elevated urine leukocytes, etc.) are reported as the name of adverse events respectively;

2. Explain as accurately as possible, such as "blockage" is where, such as the nasal cavity, lungs, liver, heart;

3. Use standard medical terms to ensure they are written correctly; do not use abbreviations to avoid slang;

4. Use the same term to describe the same event;

5. Do not use the pathogen name alone when recording the medical terms. For example, recording a bacterial infection with Staphylococcus aureus, Instead of recording Staphylococcus aureus.

Classification of disease severity (AE level):

**Mild:** The subject is tolerable, does not affect treatment, does not require special treatment, and has no impact on subject rehabilitation.

**Moderate:** the subject is intolerable, need drug withdrawal, discontinuation or special treatment, to recover the subject straight Connect the impact.

**Severe:** endanger the subject's life, death or disability, requiring immediate drug withdrawal or emergency treatment. For any serious adverse events, please fill in the serious adverse event report form and report them according to the prescribed procedures.

Classification of the correlation between adverse events and drugs

**Certain:**Use of the experimental drug has a definite relationship with time. The event is consistent with the known adverse reactions of Chinese herbal medicine. Adverse events disappear after drug withdrawal and reappear after repeated administration.

**Probable:**Use of the experimental drug has a reasonable relationship with time. Adverse events are partially in line with the known adverse reactions of Chinese herbal medicine. It is difficult to identify a cause owing to disease or other reasons.

**Possible:**Use of the experimental drug has a reasonable relationship with time. Adverse events do not conform to adverse reactions of Chinese herbal medicine. The adverse cvents are likely to be caused by diseases or other reasons.

**Remote:**There is a possible connection between time and experimental drug. It is easily explained and verified through the disease or other reasons.

**Unrelated:**There is no connection between time and the test drug. Adverse events are definitety caused by the disease or the main cause of the discase

Relationship between AE and trial drug: It is recommended to analyze the relationship between AE and trial drug according to the following five principles, whether the time of ① start has a reasonable sequence relationship with adverse events / adverse reactions; whether ② suspected ADR conforms to the known ADR type of the drug; whether the ADR suspected by ③ can be explained by the patient's pathological condition, concomitant medication, concomitant treatment or previous treatment; ④ discontinuation or suspicious dose reduction Whether ADR decreased or disappeared; ⑤ experienced the same reaction after reuse of suspect drug.
